# Applying quality improvement methods to address gaps in medicines reconciliation at transfers of care from an acute UK hospital

**DOI:** 10.1136/bmjopen-2015-010230

**Published:** 2016-06-09

**Authors:** Vanessa Marvin, Shirley Kuo, Alan J Poots, Tom Woodcock, Louella Vaughan, Derek Bell

**Affiliations:** 1 Pharmacy Department, Chelsea and Westminster Hospital NHS Foundation Trust, London, UK; 2 National Institute for Health Research (NIHR) Collaboration for Leadership in Applied Health Research and Care (CLAHRC) North West London (NWL), Imperial College London, London, UK; 3 NIHR CLAHRC NWL, Imperial College London, London, UK; 4 Department of Acute Medicine, Chelsea and Westminster Hospital NHS Foundation Trust, London, UK

**Keywords:** Medication reconciliation, Patient safety, hospital pharmacist, quality improvement

## Abstract

**Objectives:**

Reliable reconciliation of medicines at admission and discharge from hospital is key to reducing unintentional prescribing discrepancies at transitions of healthcare. We introduced a team approach to the reconciliation process at an acute hospital with the aim of improving the provision of information and documentation of reliable medication lists to enable clear, timely communications on discharge.

**Setting:**

An acute 400-bedded teaching hospital in London, UK.

**Participants:**

The effects of change were measured in a simple random sample of 10 adult patients a week on the acute admissions unit over 18 months.

**Interventions:**

Quality improvement methods were used throughout. Interventions included education and training of staff involved at ward level and in the pharmacy department, introduction of medication documentation templates for electronic prescribing and for communicating information on medicines in discharge summaries co-designed with patient representatives.

**Results:**

Statistical process control analysis showed reliable documentation (complete, verified and intentional changes clarified) of current medication on 49.2% of patients' discharge summaries. This appears to have improved (to 85.2%) according to a poststudy audit the year after the project end. Pharmacist involvement in discharge reconciliation increased significantly, and improvements in the numbers of medicines prescribed in error, or omitted from the discharge prescription, are demonstrated. Variation in weekly measures is seen throughout but particularly at periods of changeover of new doctors and introduction of new systems.

**Conclusions:**

New processes led to a sustained increase in reconciled medications and, thereby, an improvement in the number of patients discharged from hospital with unintentional discrepancies (errors or omissions) on their discharge prescription. The initiatives were pharmacist-led but involved close working and shared understanding about roles and responsibilities between doctors, nurses, therapists, patients and their carers.

**Strengths and limitations of this study**

- We recognised the importance of organisation and structure in reducing unintended discrepancies at transfer of care.
- We showed a critical relationship between discharge summary quality and junior doctor rotations. Interventions were specifically made at these key times and appear to have had a positive effect on the numbers of patients with error-free medication lists.
- Appropriate systems changes were embedded to ensure sustainability.
- Limitations in our methodology meant we are unable to show whether the decrease in errors was directly related to introduction of pharmacist-led discharge medicines reconciliation or secular trends.
- We do not know if improvements in communications had any impact on patient outcomes postdischarge from hospital.

## Introduction

Transfers between interfaces of care, especially discharge from acute hospital into the community, are recognised as high-risk transitions for the development of medicines-related problems, a leading cause of morbidity and mortality.^1^ Medication ‘continuity’ errors are frequent, involving up to 70% of inpatients on admission to hospital^2^ and contributing to avoidable readmissions.^3^ Considering that between 28% and 40% of medicines are discontinued or altered during hospitalisation,^4^ and fewer than 10% of elderly inpatients go home on the same medication as on admission,^5^ accurate communication of changes at discharge is an increasingly important contribution to patient safety and quality of care.

Medicines reconciliation, the process of identifying the most accurate list of a patient's medicines and comparing it with current prescribing, recognising any discrepancies and documenting any changes, is essential for minimising continuity errors.^6^ The elements of reliable reconciliation are at each transition in care:

- verification (of the list of current medications the patient is actually taking);
- validation (acute review noting whether to continue, alter doses, hold, or stop);
- clarification (comparing the medication list with current prescription order).^6^

Increased pharmacist involvement at admission, documentation of changes, and systems facilitating transfer of information from the general practitioner (GP) to hospital, all appear to reduce medication error.^7^ Previous local audit had revealed that though actively involved in the timely resolution of discrepancies between patients' medicines list from the GP and the hospital doctor, there was a lack of discharge communication from hospital pharmacists. In addition, the quantity and quality of information on medication changes made during hospitalisation was low; only one in 10 patients was discharged from hospital with sufficient information on their discharge summaries to enable safe ongoing prescribing. The information required was considered insufficient if one or more medicines were omitted; a stopped medicine was included erroneously or without explanation; the dose, route, course length or formulation (or change reason) was wrong or omitted; or essential monitoring information was lacking.

We recognised the need to integrate discharge reconciliation into the processes involving ward pharmacists; that is, in confirming the clinical appropriateness of prescribing during the inpatient stay and checking back to the medicines history when organising take-home medicines. Pharmacy-led reconciliation is considered a cost-effective intervention.^7^

The overall aim of this study was to provide seamless, high-quality medicines reconciliation from admission through to discharge for all patients, and improve communication with community service providers.

The objectives were to:

- reduce unintentional discrepancies in transcribing medication during admission to hospital
- improve documentation of medicines reconciliation at discharge
- improve the quality of communications regarding new and intentional changes to medication in the hospital discharge summary.

### Ethical approval

Ethics approval was not required for this work, as it was part of a service evaluation and improvement activity and not human subjects research. An ethics waiver was granted by Chelsea and Westminster Hospital NHS foundation trust (CWH) Research and Development lead.

## Methods

### Setting

The main study was conducted at an acute hospital over 18 months, from September 2011 to March 2013. A poststudy audit to check whether any improvements have been sustained was carried out during June to August 2014. The focus of the study was the Acute Assessment Unit (AAU), a 44-bed adult ward seeing an average of 25 admissions a day with a mean age of ∼61 years. These are predominantly medical patients (17% surgical admissions) discharged home or to a longer stay ward usually within 4 days. The average length of stay in hospital was 9.3 days at the time of the study. Junior doctors are responsible for documenting the patient's history on admission (including their medicines), prescribing ongoing medication and preparing the discharge summary. The pharmacist on AAU verifies the medication history, validates and checks that all current continuing medicines are correctly prescribed on the inpatient electronic prescribing system (ePR). If a discrepancy is found, or a change is made without the reason or indication documented as part of the medication order, it is clarified by the pharmacist. The prescriber is contacted to ascertain if the change was intentional. The completion of this pharmacist-led process of reliable reconciliation at admission is also documented appropriately on the ePR. Discharge prescribing is supported by pharmacists who check (or transcribe) take-home medicines (TTO). When the hospital has reduced capacity to admit to AAU, the focus for medical teams shifts to support speedier discharge including writing TTOs as early as possible. Early discharge relieves the bed pressures and allows for admission of new patients. Pharmacist activity on AAU is not usually affected by these changes and was maintained throughout the project.

### Planning the intervention

Following recognition of low overall numbers of patients whose medicines are fully reconciled, a core team of pharmacists and physicians convened with the objective of improving rates locally. Quality improvement (QI) methodologies were employed throughout.^8^ ^9^ Workshops took place at the start of the project to identify stakeholders (see online supplementary appendix figure 1), and their engagement was plotted on the matrix again at 15 months (see online supplementary appendix figure 2). Process mapping identified the various stages of medicines reconciliation in the hospital (see online supplementary appendix figure 3), and was repeated with the focus on AAU (see online supplementary appendix figure 4). For this, we convened a multidisciplinary team which included senior clinical leaders, senior nurses, junior doctors, consultant physicians, therapists, pharmacists and a data analyst. All contributed to the mapping and development of the interventions (see online supplementary appendix figure 5). For example, the physiotherapists advised on how they check a patient's use of medication compliance aids, and occupational therapists on finding ‘old’ medicines during home visits. Stakeholder engagement events open to staff and public were held, and regular patient focus groups around medicines management topics continued through to July 2012. Members of the public were called on on an ad hoc basis at first, and subsequently, patient representatives were fully recruited to the core team resulting in co-design of our interventions and systems updates. An Action Effect Diagram was drawn with contributions from all stakeholders and the overall aim agreed (see online supplementary appendix figure 6).^8^ Plan Do Study Act (PDSA) cycles further informed the project from the beginning and as it progressed (see online supplementary appendix table 1).^9^ Stakeholders received feedback through emails and personal communications when the process maps were finalised.

10.1136/bmjopen-2015-010230.supp1 Supplementary appendix

Interventions were agreed as the most likely to lead to measurable improvements, assigned into one of three work streams:

- education,
- documentation,
- communication out of hospital.

### Analytic plans

The study was a qualitative and quantitative improvement project using statistical process control (SPC) to monitor improvement measures.

SPC analyses are a graphical family of techniques designed for looking at data over time. SPC uses a number of ‘rules’ to determine whether a process has unusual variation (special causes), or if fluctuations observed are simply representative of the inherent properties of that process.^10^ In this study, we use the flexible XmR analysis and consider special causes to be indicated by points falling outside the natural process limits; a trend of six or more all increasing or decreasing values, and seven or more points consecutively above or below the mean line.^11^ Qualitative analysis of outputs from workshops, focus groups and stakeholder events, were undertaken as they took place throughout the project. Themes emerging from the analyses, including patients' wish to have their own summary of new medicines on discharge with a personalised list of side effects (rather than the full medicine package information) in plain language, were used to co-design the new style Discharge Summary (DSUM) (see later, Interventions). In addition, the early analysis helped form the structure and content of staff education and induction sessions (see later, Interventions).

Data collation was carried out each week by the research pharmacist (SK). A sample of 10 discharge prescriptions was identified weekly using randomly generated numbers. Checks were put in place to ensure that no patient was included more than once; readmissions were identified and noted (but not analysed for this project). Data was obtained retrospectively from ePR and dispensing records to identify any unintentional discrepancies between the inpatient prescription chart and discharge list of medicines. Confirmation of pharmacist-led verification of a patient's medication history was obtained from documentation in the electronic pharmaceutical care notes and the discharge summary for admission and discharge, respectively.

Process measures were designed to monitor improvements (see table 1).

**Table 1:** Process measures

Measure	Measure in sample of 10 patients per week randomly selected from all discharges for the week	Detail
1	Percentage of patients with pharmacist-verified reconciliation on admission	Pharmacist has documented on ePR that they have checked the admission medication list with the patient and verified with a second source and clarified or resolved any discrepancies on the inpatient order with the prescriber
2	Percentage of patients with pharmacist-verified reconciliation at discharge out of the total number of patients sampled	Reconciliation at discharge is possible only for patients with a verified admissions medication list. For this measure, any change to any admission medicine, dose, frequency or route is confirmed by a pharmacist as intentional and documented clearly on the discharge summary as such
3	Percentage of patients with error-free TTO prescriptions	TTO has no unexplained discrepancy compared with the verified list of medicines on admission. The reason is stated for any omission, change in dose, frequency or route; course lengths and monitoring advice are given where needed. If no reason is given for a discrepancy then the patient does not have an error-free prescription
4	Percentage of medications unreconciled at discharge out of the total number of medicines within the sample of 10 discharge summaries per week	Measure 4 is directly related to measure 3. The number of individual medicines unreconciled were recorded. Patients on no medicines were included in the study; medicines reconciliation was considered reliable only if ‘nil regular medication’ was verified and documented as such
5	Percentage of medications with an error (or omission) on TTO out of the total number of medicines within the sample of 10 discharge summaries per week	Measure 5 is directly related to measure 3. The number of individual medicines with an error or omitted without explanation were recorded. For each patient, several medicines may be prescribed in error or omitted from the TTO

ePR, electronic prescribing system; TTO, take-home medicines

An error was recorded if any medicine was ordered that should have been stopped (including wrong medicine) or if a dose, route, course length or formulation was incorrect. An omission was any medicine left off the TTO that should be entered as it is to be continued. Any change from the verified admissions list of medicines without explanation or monitoring requirement was also considered an error.

Weekly analysis of these measures was facilitated through the web improvement support for healthcare (WISH) tool.^12^ The tool provides reports with SPC analyses, by calculating the mean and respective upper and lower natural process limits of the measures in question, tracked over time. Results were fed back to the core project team weekly.

The improvement measures supported the iterative changes during implementation process and the use of PDSA cycles, also documented through the WISH software. Several audits measuring standards of medicines history taking and reconciliation of discrepancies were undertaken during the study period and helped to inform and support the project. Further details of QI methodologies and outputs are given in the online supplementary appendices.

Data were collected from patients discharged between weeks starting 30 October 2011 and 17 February 2013 (70 weeks, with one missing week). A poststudy audit was carried out using the same sampling method from 06 June to 31 August 2014 (9 weeks), to check whether any improvements made during the project were sustained. Small variations in selected numbers occurred in-week where there were delays in a patient's discharge. These patients were not excluded but appeared at a later date in the measures data.

### Interventions

All interventions took place during October 2011 to February 2013. Further details are provided in the online supplementary appendices.

#### Education

All pharmacists and medicines management technicians received a training update and accreditation in medicines reconciliation and were instructed in the importance of full documentation of preadmission medication histories. Feedback was provided on a regular basis, at least twice monthly, advocating ‘good practice’ in summarising changes made to medication during hospitalisation. Training was held collaboratively with other staff groups including nurses and therapists.

The team negotiated with AAU physicians to take a 10-min ‘Pharmacy session’ on AAU during the weekly ‘learning at lunch’ for doctors. At these sessions, and also at induction, around midyear changeover (November/December and March/April), and before end-of-year change (July/August), a pharmacist describes the principles of medicines reconciliation, good prescribing and monitoring. They also advise on timely administration of critical medicines, reviewing and continuing regular medication, and how pharmacists support the processes involved.

Two junior doctor champions were recruited to assist with the delivery of training and act as a channel for providing feedback to their peers. The project champions were well received (informal feedback from peers), and reported high levels of satisfaction with their role (informally direct to the rest of the project team and at appraisal with their clinical leads).

#### Documentation

ePR Provides an easily accessible central documentation of patients' current medication and relevant history including what the patient actually takes, their allergies, intolerances and preferences, on the same screen as inpatient prescribing. This allows access to the original list while prescribing so that changes made by the hospital clinicians can be transcribed onto the discharge documentation with ease. However, locally the medication history list and medicines reconciliation detail required free-typing, without a set format or obligatory fields. Following consultation with IT support and the junior doctor champions, changes to the system were designed by the project team and approved by the executive lead for ePR, creating tools to prompt and aid documentation of medication reconciliation. (These were brought in during the project data collection period in October 2012 as an intervention, so that we are able to measure any effect on documentation and communication) and included

- Changing screen colours to distinguish between reconciled and unreconciled medication lists.
- Changing existing ‘Pharmacy Discharge Summary Text’ box visible on GP, patient and pharmacy copy to ‘Pharmacy Screening/Dispensing Text’ only visible on pharmacy copy. GPs and patients previously received unnecessary dispensing information on their discharge summary.
- Creating a ‘Pharmacy Medicines Management Text’ box, to allow clear timely documentation by pharmacists of medicines reconciliation, and information about changes visible as required on all copies. This includes confirming where medicines reconciliation was not completed at admission.
- The addition of space headed ‘Information for Patient’ on the patient copy of the discharge summary for the pharmacist to add selected counselling points specific to their new medicines.
- Signposting to the hospital medicines information helpline to aid access to further information they may need once they are home, developed in response to patient experience feedback.^13^ ^14^

#### Communication with the GP

At first presentation at hospital, an individual patient's complete list of current medication is required either via the patient or their carer (eg, a repeat prescribing document or detail on a referral letter from the GP), or if this is not with the patient, the GP surgery is usually contacted at the earliest opportunity. There is as yet no direct e-communication locally between the hospital ePR and GP practices. We use the telephone to request and fax to receive patient medication record details. On transfer home, we create the discharge summary including the TTO which is for many medicines, a simple transfer from the inpatient ePR. A copy is emailed or posted to the GP. Communication out to the GP about any changes made to medication in hospital requires free-typing into the discharge summary; local audit found this was missing in over 40% of cases. The approved changes to the ePR documentation as above were designed to improve medication reconciliation communication, including with the GP.

## Results

**Figure 1:**
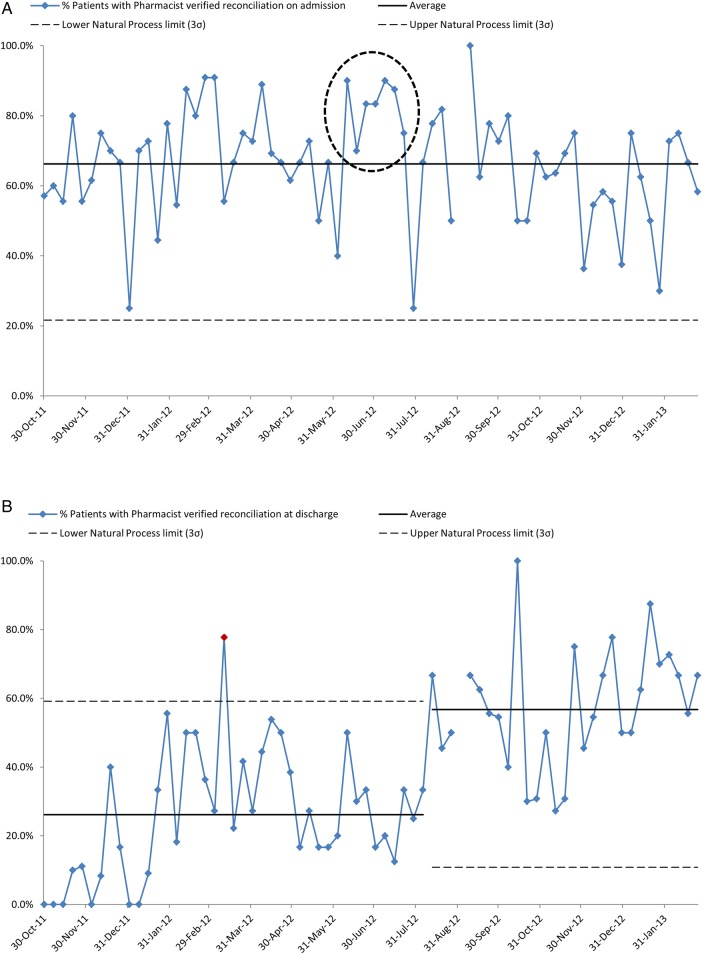
(A) (Measures 1: higher percentage preferred): percentage of patients with pharmacist-verified reconciliation on admission. (B) (Measure 2: higher percentage preferred): percentage of patients with pharmacist-verified reconciliation at discharge.

**Figure 2:**
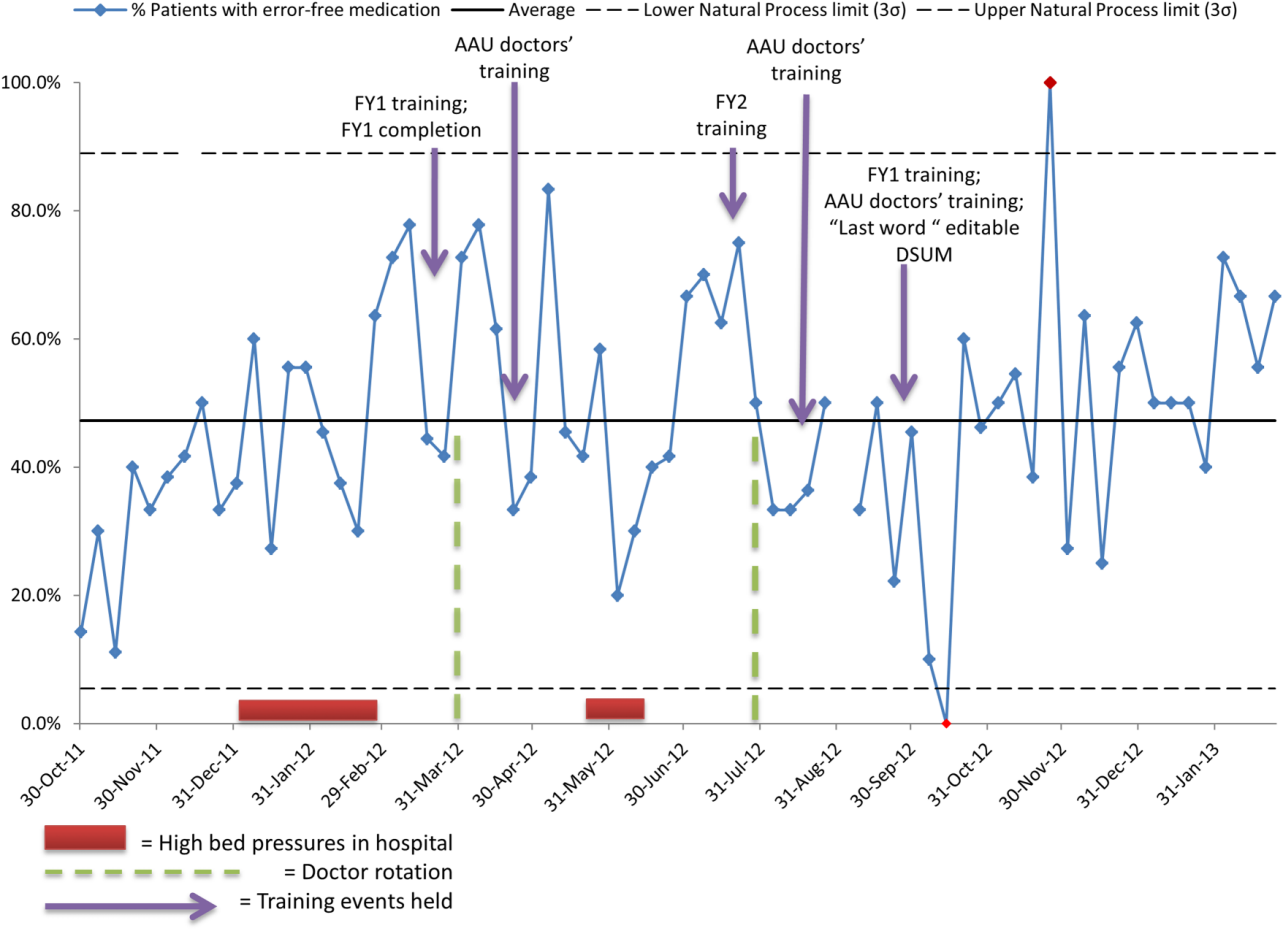
(Measure 3: higher per cent preferred): percentage of patients with error-free (and no omitted) medications on TTO prescriptions\r\nKey AAU, Acute Admissions Unit; DSUM, Discharge Summary; FY, foundation year junior doctors; ‘Lastword’: the local EPR system.

**Figure 3:**
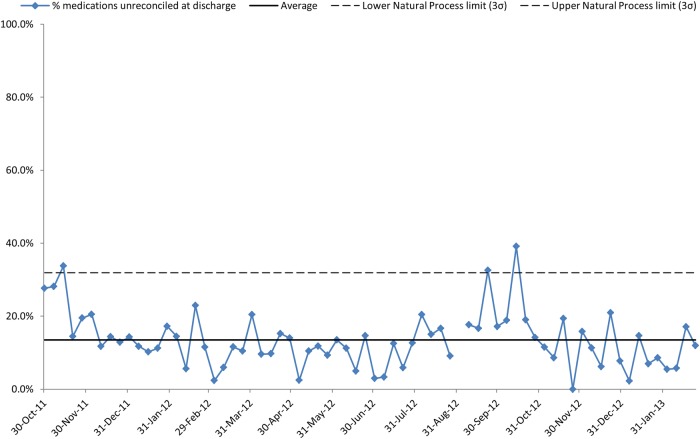
(Measure 4: lower per cent preferred): the percentage of medications unreconciled at discharge.

**Figure 4:**
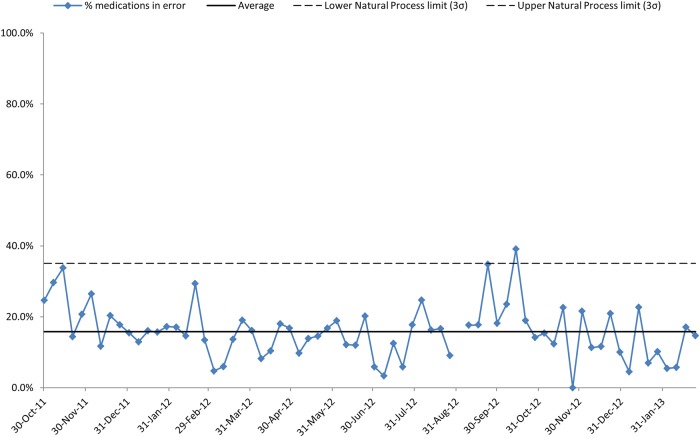
(Measure 5: lower per cent preferred): the percentage of medications with an error or omission on TTO.

A step-wise improvement is seen across measures relating to discharge medicines reconciliation throughout the project (figures 1–4). For the poststudy audit, all measures indicate sustained improvement, summarised in table 2.

**Table 2:** Audit data to examine for sustainability of changes

For audit period: weeks starting 6 July 2014 to 31 August 2014				
Number of patients in audit=88, number of medications=1148, mean number per patient=13				
% Patients with pharmacist-verified reconciliation on admission	% Patients with pharmacist-verified reconciliation at discharge	% Patients with error-free medication	% Medications unreconciled at discharge	% Medications in error
87.5	64.8	85.2	3.7	2.3

During the study period, an average of 66.3% of patients have pharmacist-verified medicines reconciliation on admission (see figure 1A). A temporary uplift in the process is observed starting in June 2012 with seven points above the mean line, however, the process reverts to previous performance levels after this period. The average (mean) showed some short-term improvement to 82.7% coinciding with when initiatives were put in place to engage staff in pharmacist-led processes. Reconciliation at discharge is possible only for those who had a verified list of admission medicines.

Pharmacist documentation of medicines reconciliation at discharge improved from an average of 26.2% of patients to 56.7% (figure 1B). A single point outside the natural process limits is observed in March 2012, indicating a special cause. From August 2012 onwards, all points lie above the previous mean performance (special cause variation), hence the natural process limits are calculated separately for this period to better represent the improved process. This improvement appears to be sustained and improved on, as it was found during the summer of 2014, that an average of 64.8% of discharged patients had their medicines reconciled and documented on the discharge summary (table 2).

On 1 week with high bed pressures (31 May 2012, see figure 2) performance was below average, recovering over a 6-week period of increasing trend (constituting an SPC rule break). There are two indications of special cause with data lying beyond the natural process limits in October 2012 and November 2012. The periods of bed pressures did not appear to affect pharmacists' admission activity.

The short-term improvement mid-project appears to have been achieved 1 year on as it was found that in a 9-week period of measures during summer 2014, an average of 88.1% of patients had pharmacist-led medicines reconciliation documented on admission.

After an initial low period, an average of 47.2% of patients with no medication errors or omissions on discharge is seen, but with marked variation in late 2012 coinciding with the changes being embedded in the editable part of the discharge summary (figure 2). In the period of measures during summer 2014, an improvement was seen with 85.2% patients having error-free medication using the same criteria for reconciliation as during the project 18 months previously (table 2).

Key events mapped onto the process control chart for error-free medications from admission and through to discharge during one calendar year of the project, show the relationship between junior doctor rotations and the weeks when the hospital was under bed pressures (figure 2). A fall in the percentage of error-free medications is seen during September 2012 though this is not sustained and improvements are apparent when teaching sessions had been completed.

The average for medications unreconciled was 13.5% (figure 3). There are three indications of special cause, October 2011, September 2012 and October 2012. In the summer of 2014, improvement was found with 3.7% of medicines recorded as unreconciled at discharge (table 2).

The percentage of medications with an error (or omission) was an average of 15.8% (figure 4). There are two indications of special cause variation, September 2012 and October 2012. During summer 2014, improvement was seen with an average of 2.3% of medicines (prescribed or omitted) in error using the same criteria as during the project (table 2). Note that in figures 3 and 4 there are transient uplifts in values, before reversion to previous performance, across August and September 12; the period in which newly qualified doctors begin their training.

## Discussion

Hospital-based, pharmacist-led medicines reconciliation processes frequently identify and resolve unintended prescribing discrepancies between healthcare providers.^1^ We have made improvements to these local processes particularly in provision of documentation and communication of medication changes at discharge from hospital.

The effect of this QI is demonstrated in the decrease in numbers of patients leaving hospital with unintentional discrepancies (errors or omissions) on their discharge prescription. Though there was marked variation in this figure during the study, it appears to be sustained overall with an expectation that it remains consistently below 20% (as shown in 2014). However, the period from August to October in 2012 shows an increase in the number of unreconciled discrepancies in discharge medications. We have looked for explanations for this as it does not coincide with the hospital being particularly busy or under pressure for beds or other parameters that we were monitoring at the time. It may have been influenced by the period of high staff turnover in pharmacy which occurs every new academic year. Though not the project team per se, we were inducting new juniors and managing unprecedented vacancies including staff leave (postponed during the London Olympics and taken in September and October that year).

There is clearly a need for further improvement; regular teaching and support, particularly for junior doctors, have been put in place, and remain a key aspect of current practice and the subject of further medicines optimisation research locally. In addition, the pharmacist induction programme locally now includes training in documentation of medicines reconciliation on ePR.

We found a high level of variation in the percentage of patients with error-free discharge prescriptions, in particular, around the time of introducing the changes to processes on ePR. The changes required different inputs by the prescriber, and though all were trained by the implementation date, many had their training several weeks before. Variations may also have been the result of the small sample set for weekly measures. Ten patients were selected each week. If a fully trained ‘good’ prescribing team were on duty for the sampling period it could contrast with one less familiar with TTO requirements on duty the following week.

Overall, our ePR updates appear to have had a positive effect on the quality of discharge summaries, as error-free TTO rates are seen to rise in the period from its inception in October 2012 to February 2013 when measurement stopped, and again when measured in 2014.

A median of 45% of hospital patients in the USA and Canada have at least one clinically significant discrepancy in their medications at transfer of care according to a systematic review of reconciliation in 2013.^15^ Garfield and colleagues in the UK found unintentional discrepancies in 70% of medication prescribed on admission for around 60% of patients.^16^ Unintentional discrepancies in discharge medication received by patients occurred in up to 27% of items, and these translated to discrepancies in repeat medication subsequently received from the GP in 57% patients.^17^ In our study, we looked at documentation on the discharge summary exactly as it would be received by the GP. An ‘error’ was recorded if a medicine was missing from this communication, or if details of a change in medication were not noted. The number of medicines unreconciled at discharge fell to 10% and then to 4% (2014 figures). Ascertaining whether any changes to medication reported are actually received and acted on by the recipient was outside the scope of this project.

Follow-up of patients at another UK hospital where medicines reconciliation was found to be incomplete revealed that the majority of failures occur when the standard admission documentation is not used. This was more likely to occur where specialist admission pathways were in place and paper pro formas were not updated, or if they had to be used in parallel with several other documents.^17^ A survey of pharmacy services for patients at discharge from hospitals in Ireland suggested that development of national standards of practice may help to eliminate the variation found in practice and would support improvement.^18^ During our study, we embedded new ePR tools to prompt and aid documentation of medication reconciliation particularly on the discharge summary. In addition, at admission, we sought to standardise the pharmaceutical care entries made by pharmacy staff regarding medication histories. An audit undertaken in 45 English hospitals (including this study site) suggests that pharmacist-led medicines reconciliation at admission prevents adverse events occurring during an inpatient stay.^19^

In the 2013 systematic review, the authors note that the actual benefits of resolving unintended discrepancies are not seen; medicines reconciliation does not seem to reduce emergency department visits or readmission within 30 days. The reviewers found that most medication discrepancies appeared to have no clinical significance and, given limited resources in hospitals, it is suggested it may be prudent to target patients at high risk rather than all admissions.^15^ Our study did not include patient follow-up, so does not add to this, but follow-on projects are planned where we will target vulnerable patients (especially elderly) identified through medicines reconciliation and other processes for further pharmacist intervention with examination of the clinical significance of intervening on unintentional discrepancies and readmission rates.

In part, to inform this research, we recently compared medicines reconciliation by doctors on first contact with patients to pharmacy-verified medication lists. Full and accurate documentation was found for only 27% of patients prior to pharmacy check. The value of the pharmacist in medicines reconciliation was also shown in a Swedish medical ward though the researchers suggested more work is needed.^20^

Documentation by pharmacists of medicines reconciliation at discharge in addition to that undertaken on admission was a new concept locally. We have now integrated the process into the patient-centred pharmaceutical care carried out by our team of clinical (ward) pharmacists as part of their regular duties. All inpatient prescriptions are reviewed by a pharmacist at the first opportunity, including medicines reconciliation within 24 hours of admission where possible. It is a challenge at weekends where staffing levels are lower; currently under review locally and across the UK. The changes we have put in place around discharge reconciliation have been achieved without extra resources but with critical refocussing of pharmacist input. Prior to this project, any changes made to patients' medicines had to be communicated by the prescriber as part of the free-type letter to the GP on the discharge summary.

There appears to be a relationship between quality of discharge summary and junior doctor rotations. Interventions specifically made at key times in rotations to improve discharge summary documentation appear to have a positive effect on the number of patients with error-free TTOs.

We recognised the importance of organisation and structure in reducing unintended discrepancies at transfer of care. A ‘whole system’ approach in this discharge process involved members of staff from a range of disciplines, all of whom were involved in appropriate prescribing, ensuring the assessment of a patient's ability to take their medication, or education of a patient about their discharge medications. While other studies have underlined the importance of the interactions between medical and pharmacy staff, the success of this project partly lay in its ability to engage with nursing and allied health staff in addition.

The project team made ongoing sustainability a priority from the start, which is judged as important in embedding change,^21^ and where appropriate, systems change was sought (eg, improved electronic prescribing software functionality). Building improvements into the processes helps to minimise human error and reduce variability of outcomes. Better use of existing resources, and embedding new tools for daily practice therein, ensures a sustainable change for the organisation which might be expected to be cost-neutral.

Integration of best practice project management using QI methods ensured a clear structure to the project organisation and management, while allowing room for creativity.

## Limitations and lessons learnt

We were unable to show if our improvements in communication out of hospital had any impact postdischarge. This will be the subject of future project work in the community. The data presented here suggests a link between pharmacist involvement and a decrease in errors, but is not conclusive. We were not able to examine for secular trend as there are no prior data or further sites; we recommend a step-wedge design for any scale-up initiative to allow comparisons.

The project team was successful in engaging and influencing staff from all levels in changing practice. Communication barriers with doctors where they existed were removed with the recruitment of junior doctor champions to deliver training and provide feedback to peers. Culture within the pharmacy department was changed by seeking out early adopters to act as catalysts for change. Engaging the right people at the right time for the right tasks that complement their skills and interests, was a key to success (eg, AAU sister in mapping discharge process; junior doctors in preparing posters).

This included effective engagement with the hospital's GP Relationships Manager who supported the project's initiatives where possible; this proved important, as engaging directly with GPs was difficult.

Other aspects of the project, such as junior doctor and patient education, which are labour intensive, were successful but may prove less sustainable.

## Recommendations

Regular feedback of the quality of doctor's medication reconciliation at discharge is an important aspect of training that has resulted in an improvement in the number of patients discharged without errors on the discharge summary. However, maintaining weekly measures to allow such feedback is very time consuming. An option could be through incorporating the weekly measures into Trust clinical audit agenda.

The data in the current form are unable to distinguish whether the improvement in number of unreconciled medicines or number of errors is because of the introduction of pharmacist discharge medicines reconciliation and documentation. We do not know if they resulted in improved patient outcomes nor if communications in the discharge summaries are actioned by the recipient. We therefore recommend that a subset analysis and follow-up is carried out to compare outcomes for patients who have had pharmacist involvement in the preparation of the discharge summary.

## Conclusion

During the period of our medicines reconciliation project we put in place new processes that led to a sustained reduction in unreconciled medications and, thereby, an improvement in the number of patients whose discharge medications were documented and communicated out from the hospital without error or omission. The initiatives were pharmacist-led but involved close working and shared understanding about roles and responsibilities between doctors, nurses and patients or their carers.

Care has been taken to embed the processes involved into standard working practices and computerised systems, ensuring that reliable reconciliation and documentation is sustainable.

## References

[1] Royal Pharmaceutical Society. Keeping patients safe when they transfer between care providers—getting the medicines right. Royal Pharmaceutical Society, London: 2012.

[2] National Institute for Health and Clinical Excellence (NICE) and National Patient Safety Agency. Technical patient safety solutions for medicines reconciliation on admission of adults to hospital. PSG 001 Reference number 1035, issue date 1 Dec 2007 http://www.sefap.org/media/upload/arxius/formacion/aula_fap_2010/bibliografia/NHS_Technical_patient_safety_solutions.pdf.

[3] Witherington EM, Pirzada OM, Avery AJ Communication gaps and readmissions to hospital for patients aged 75 years and older: observational study. Qual Saf Health Care 2008;17:71–5. 10.1136/qshc.2006.020842 18245223

[4] Thompson-Moore N, Liebl MG Health care system vulnerabilities: understanding the root causes of patient harm. Am J Health Syst Pharm 2012;69:431–6. 10.2146/ajhp110299 22345422

[5] Mansur N, Weiss A, Beloosesky Y Relationship of in-hospital medication modifications of elderly patients to post discharge medications, adherence and mortality. Ann Pharmacotherapy 2008;42:783–9. 10.1345/aph.1L070 18445704

[6] Institute for Healthcare Improvement (IHI). Medicines Reconciliation at All Transitions. 2009 http://www.ihi.org/imap/tool (accessed 21 Aug 2013).

[7] Karnon J, Campbell F, Czoski-Murray C Model-based cost-effectiveness analysis of interventions aimed at preventing medication error at hospital admission (medicines reconciliation). J Eval Clin Prac 2009;15:299–306. 10.1111/j.1365-2753.2008.01000.x 19335488

[8] Reed JE, McNicholas C, Woodcock T Designing quality improvement initiatives: the action effect method, a structured approach to identifying and articulating programme theory. BMJ Qual Saf 2014;23:1040–8. 10.1136/bmjqs-2014-003103 25319412

[9] Taylor MJ, McNicholas C, Nicolay C Systematic review of the application of the plan-do-study-act method to improve quality in healthcare. BMJ Qual Saf 2014;23:290–8. 10.1136/bmjqs-2013-001862 PMC3963536 24025320

[10] Provost LP, Murray S The Health Care Data Guide: Learning from data for improvement. San Francisco: Jossey-Bass, 2011.

[11] Urdhwareshe H Six sigma for business excellence: approach, tools and applications. Chennai, India: Dorling Kindersley, 2011:259; ISBN: 978-81-317-3154-3.

[12] Curcin V, Woodcock T, Poots AJ Model-driven approach to data collection and reporting for quality improvement. J Biomed Inform 2014;52:151–62. 10.1016/j.jbi.2014.04.014. 24874182 PMC4266541

[13] Marvin V, Park C, Vaughan L Phone calls to a hospital medicines information helpline: analysis of queries from members of the public and assessment of potential for harm from their medicines. Int J Pharmacy Practice 2011;19:115–22. 10.1111/j.2042-7174.2010.00081.x 21385242

[14] Marvin V, Kuo S, Vaughan L How can we improve patients’ knowledge and understanding about side-effects of medicines? Storyboard Presented at the Institute for Healthcare Improvement National Forum on Quality Improvement in Healthcare; Florida, USA, December 2012.

[15] Kwan JL, Lo L, Sampson M Medication reconciliation during transitions of care as a patient safety strategy. A systematic review. Ann Intern Med 2013;158:397–403. 10.7326/0003-4819-158-5-201303051-00006 23460096

[16] Garfield S, Barber N, Walley P Quality of medication use in primary care—mapping the problem, working to a solution: a systematic review of the literature. BMC Med 2009;7:50 10.1186/1741-7015-7-50 19772551 PMC2758894

[17] Ashley M How can effective medicines reconciliation be achieved? Pharm Manag 2010;26:3–7.

[18] Grimes T, Duggan C, Delaney T Pharmacy services at admission and discharge in adult, acute, public hospitals in Ireland. Int J Pharmacy Practice 2010;18:346–52. 10.1111/j.2042-7174.2010.00064.x 21054595

[19] Dodds LJ Pharmacist contributions to ensuring safe and accurate transfer of written medicines-related discharge information: lessons from a collaborative audit and service evaluation involving 45 hospitals in England. Eur J Hosp Pharm 2014;21:150–5. 10.1136/ejhpharm-2013-000418

[20] Bahrani L, Eriksson T, Höglund P The rate and nature of medication errors among elderly upon admission to hospital after implementation of clinical pharmacist-led medication reconciliation. Eur J Hosp Pharm 2014;21:156–60. 10.1136/ejhpharm-2013-000403

[21] Doyle C, Howe C, Woodcock T Making change last: applying the NHS institute for innovation and improvement sustainability model to healthcare improvement. Implement Sci 2013;8:127 10.1186/1748-5908-8-127 24160758 PMC3827618

