# Supplementary material for: Applying quality improvement methods to address gaps in medicines reconciliation at transfers of care from an acute UK hospital
Source: BMJ Open. 2016 Jun 9;6(6):e010230. doi: 10.1136/bmjopen-2015-010230 (PMC4908889; doi:10.1136/bmjopen-2015-010230)
Supplement: Supplementary appendix [file bmjopen-2015-010230supp.pdf]

Appendix

Figure 1. Stakeholder Management Matrix – baseline

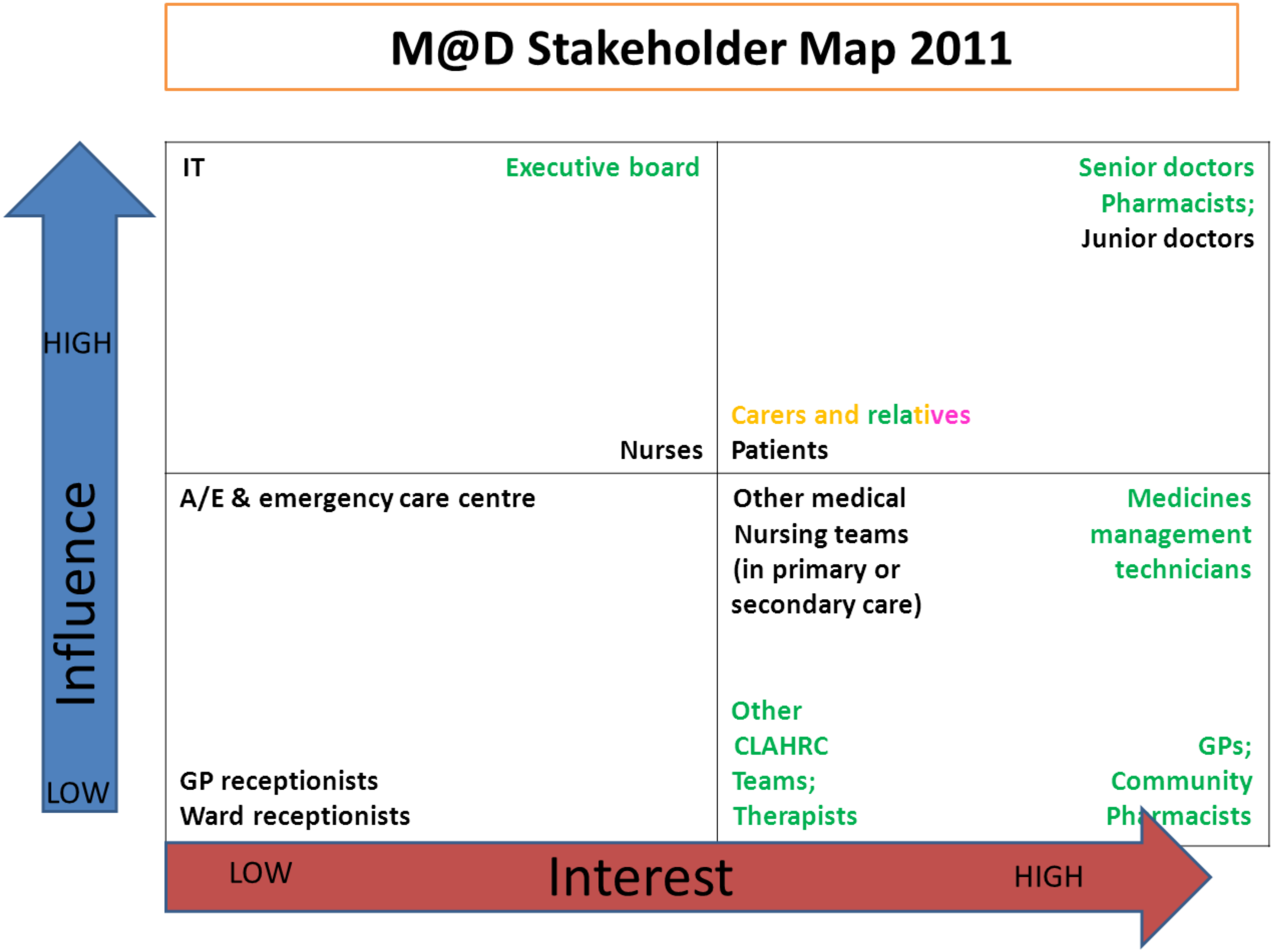

Figure 2. Stakeholder Management Matrix – 15 months

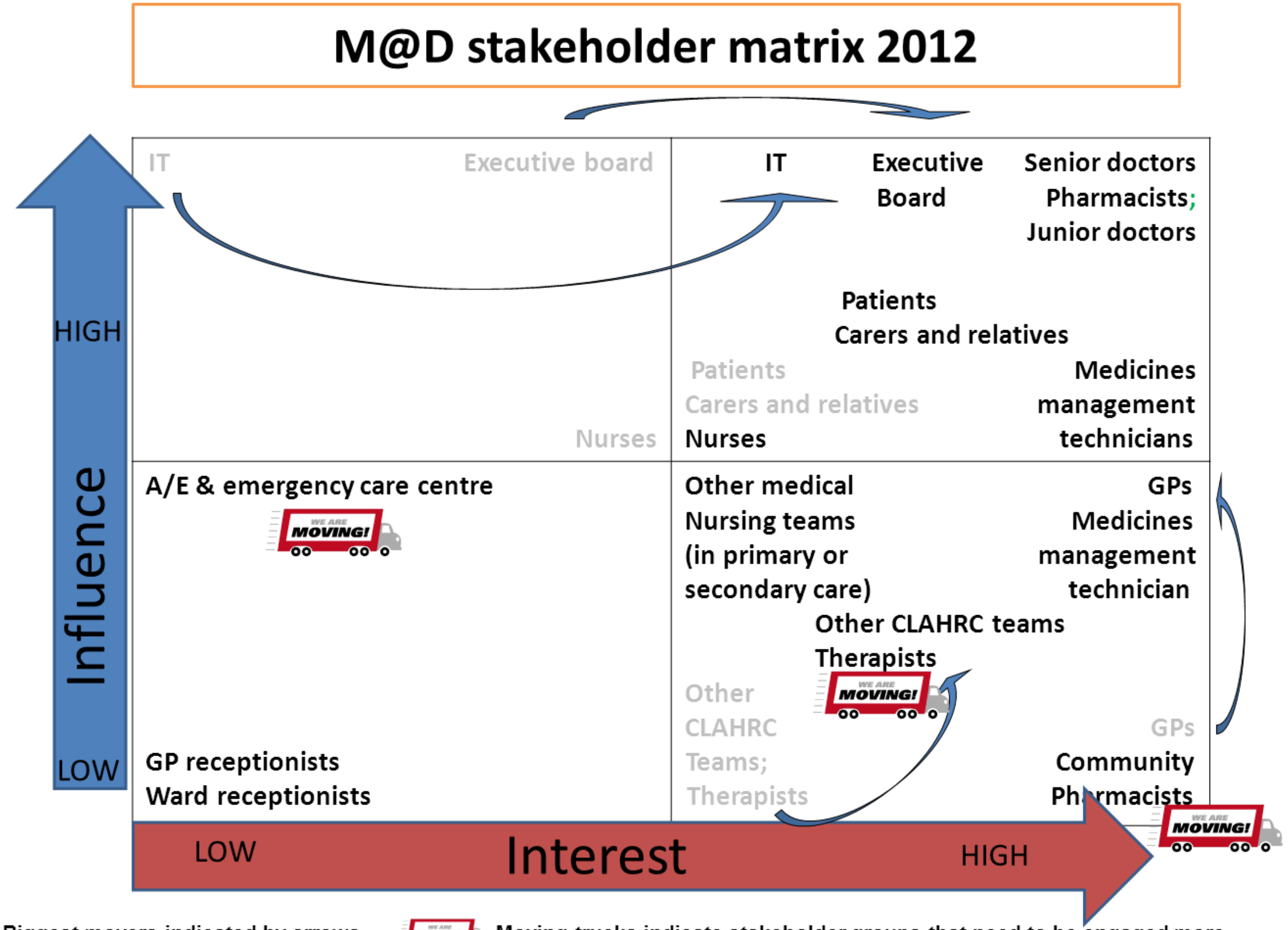

Biggest movers indicated by arrows. Moving trucks indicate stakeholder groups that need to be engaged more.

Figure 3. Process map – higher level

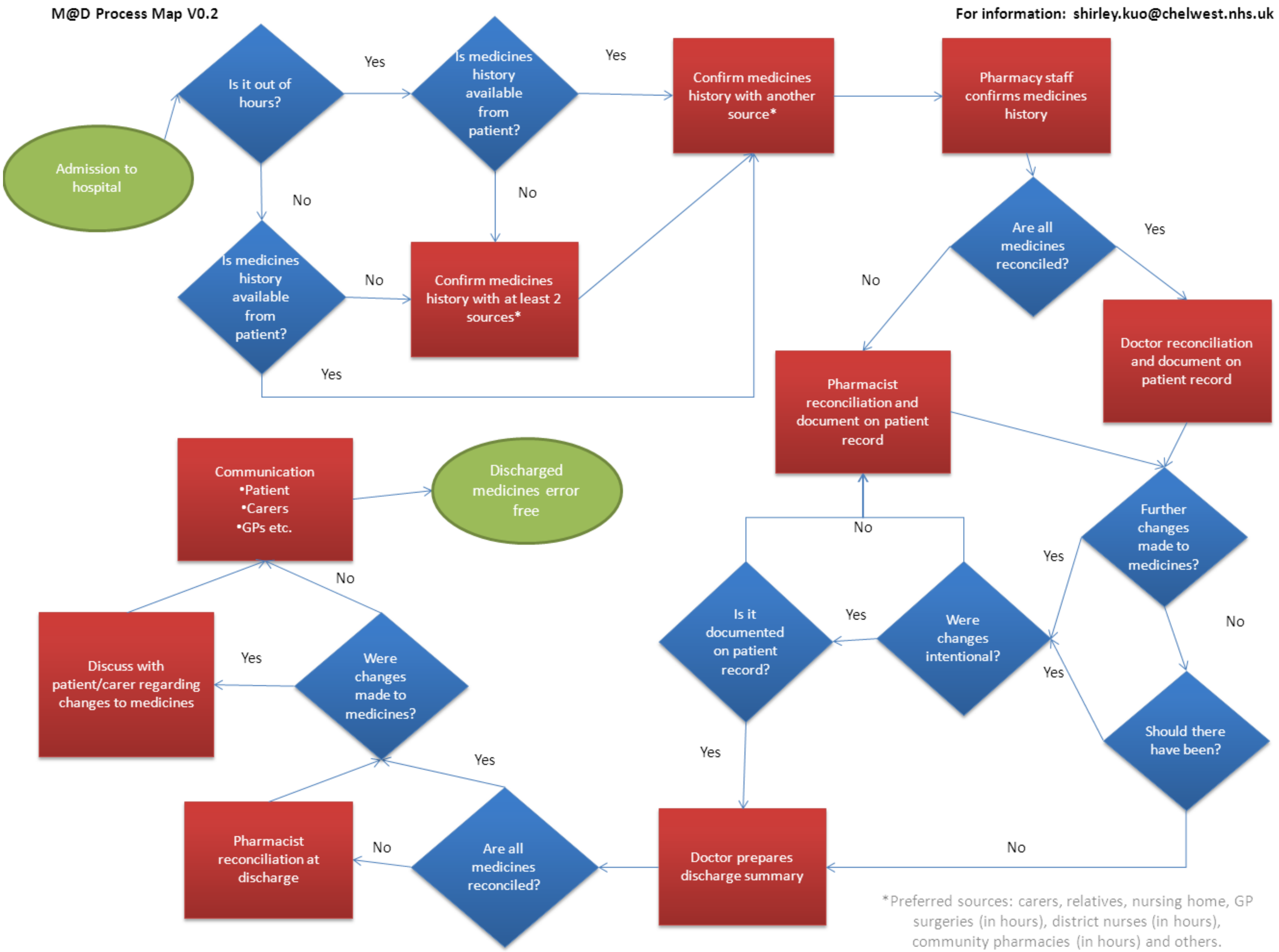

*Preferred sources: carers, relatives, nursing home, GP surgeries (in hours), district nurses (in hours), community pharmacies (in hours) and others.

Figure 4. Process map – lower level (AAU)

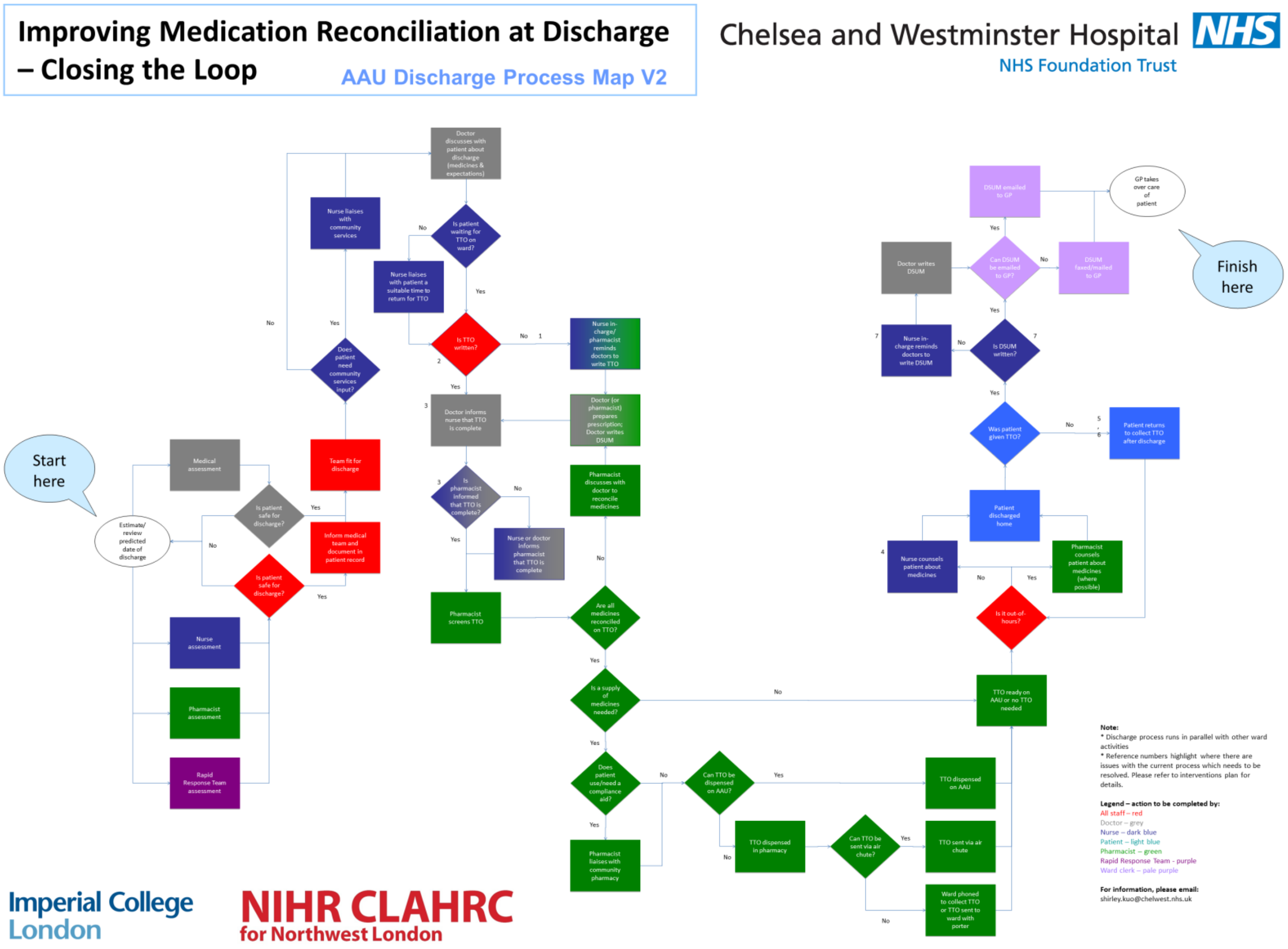

Figure 5. Embedded interventions and stakeholder groups

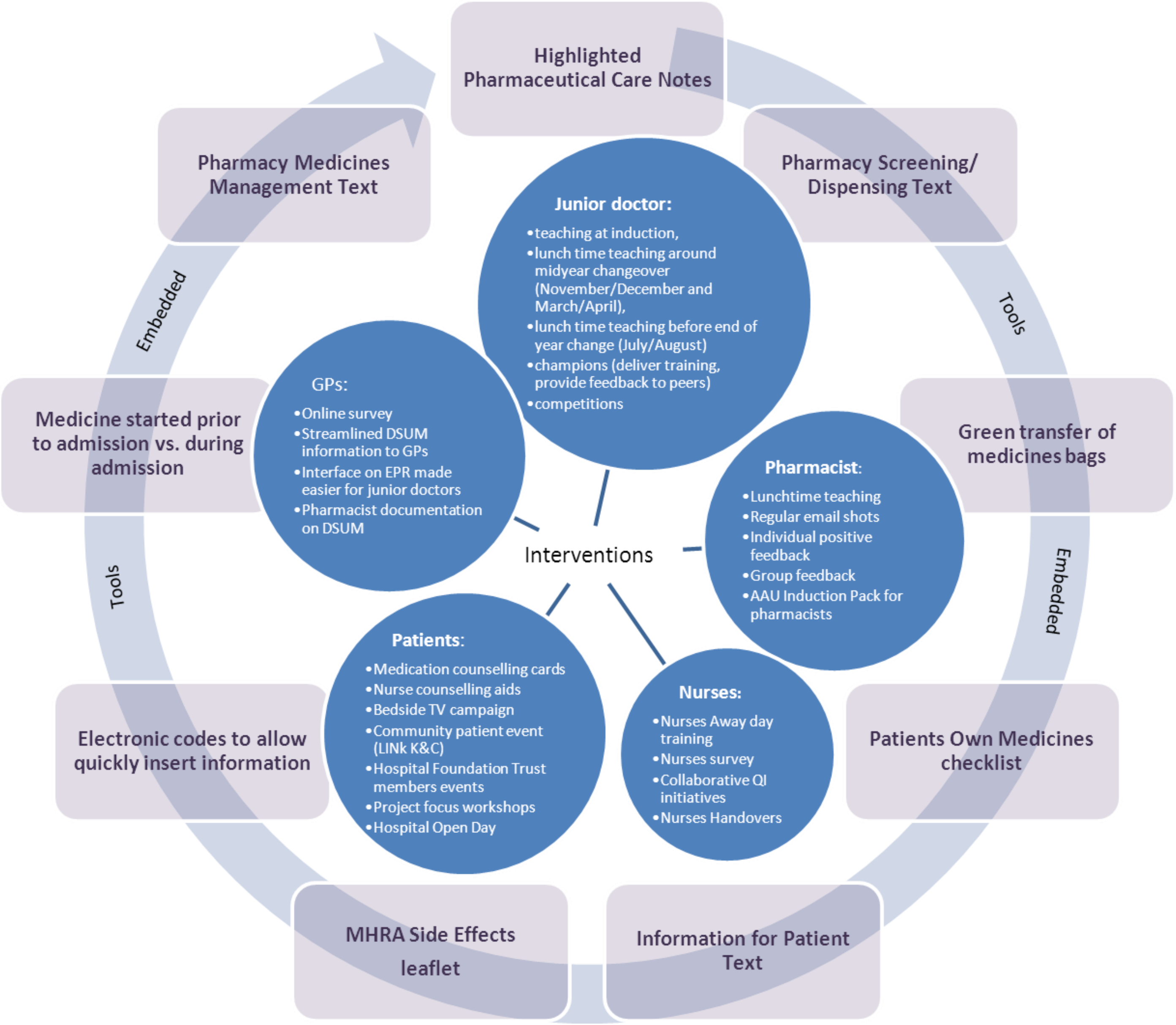

Figure 6. Action Effect Diagram

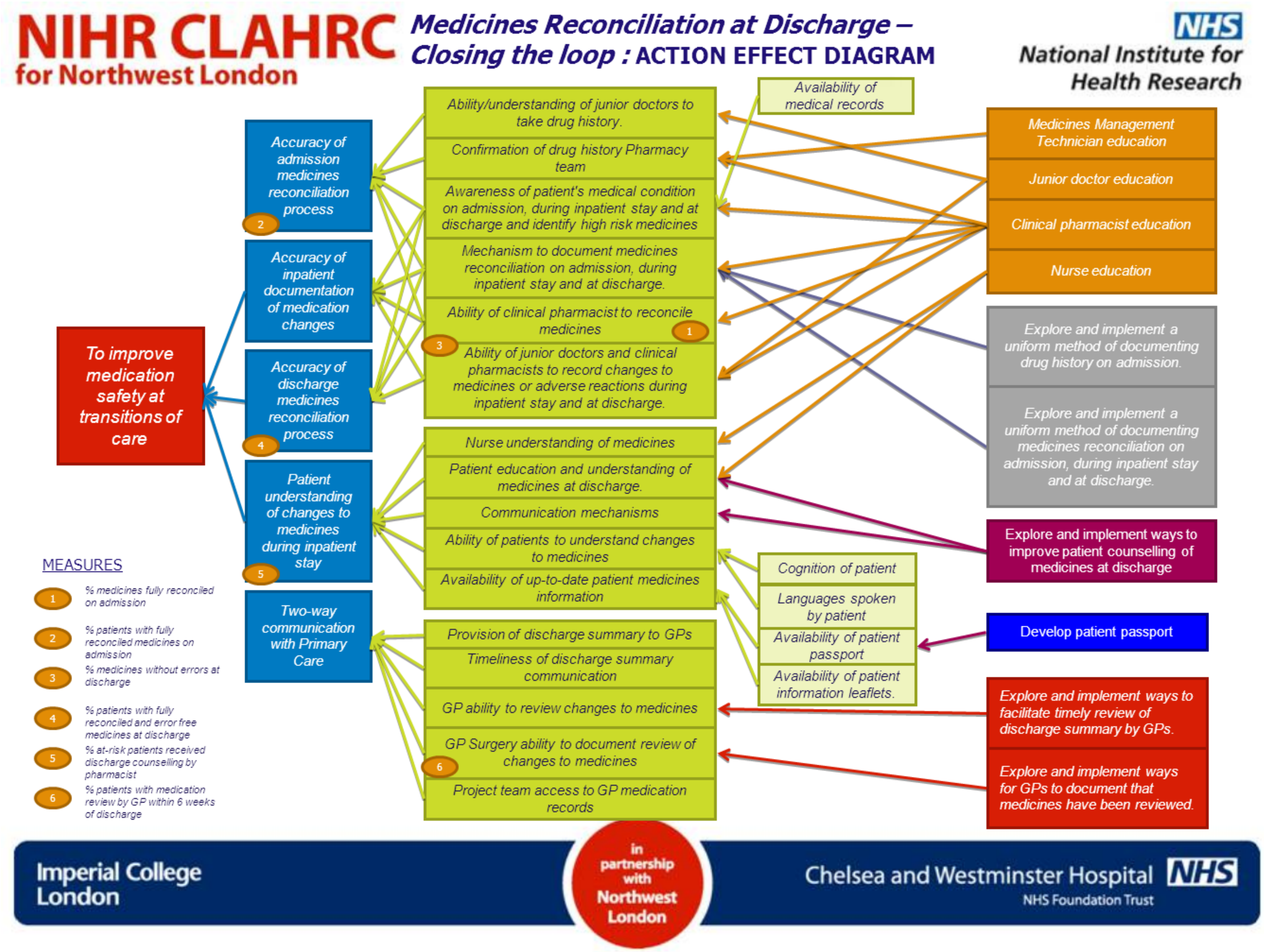

Appendix Table 1. Plan-Do-Study-Act Cycles

Series Number	Cycle title
Series 1a: 19/5/11	Transfer of medicines from AAU
Series 1b: 27/5/11	Transfer of medicines from AAU
Series 1c: 8/7/11	Transfer of medicines from AAU - Downstream wards
Series 1d: 22/9/11	Transfer of medicines from AAU - Nurses survey
Series 2: 21/5/12	TTO turnaround time - Pharmacist tracking
Series 3a: 6/12/11	Uncollected TTOs
Series 3b: 1/5/12	Uncollected TTOs
Series 4a: 11/2/12	Nurse training package - Nurse questionnaire (AAU)
Series 4b: 27/2/12	Nurse training package - Nurse questionnaire (Trust-wide)
Series 5: 28/5/12	AAU pharmacy process review
Series 6: 3/9/12	GP survey
Series 7: 14/6/12	Improvement measures

Key:
AAU = Acute Admissions Unit
TTO = To take out (medicines)
GP = General Practitioner
